# Scalability and stacking of self-stratifying microbial fuel cells treating urine

**DOI:** 10.1016/j.bioelechem.2020.107491

**Published:** 2020-06

**Authors:** Xavier Alexis Walter, Carlo Santoro, John Greenman, Ioannis A. Ieropoulos

**Affiliations:** Bristol BioEnergy Centre, Bristol Robotics Laboratory, T-Block, Frenchay Campus, University of the West of England (UWE), Bristol BS16 1QY, United Kingdom

**Keywords:** Microbial fuel cell, Urine treatment, Scaling-up, Self-stratification, Power generation

## Abstract

The scalability of Microbial fuel cells (MFCs) is key to the development of stacks. A recent study has shown that self-stratifying membraneless MFCs (S-MFCs) could be scaled down to 2 cm without performance deterioration. However, the scaling-up limit of S-MFC is yet unknown. Here the study evaluates the scale-up height of S-MFCs treating urine, from 2 cm, 4 cm to 12 cm high electrodes. The electrochemical properties of the S-MFCs were investigated after steady-states were established, following a 70-days longevity study. The electrochemical properties of the 2 cm and 4 cm conditions were similar (5.45 ± 0.32 mW per cascade). Conversely, the 12 cm conditions had much lower power output (1.48 ± 0.15 mW). The biofilm on the 12 cm cathodes only developed on the upper 5–6 cm of the immersed part of the electrode suggesting that the cathodic reactions were the limiting factor. This hypothesis was confirmed by the cathode polarisations showing that the 12 cm S-MFC had low current density (1.64 ± 9.53 µA cm^−2^, at 0 mV) compared to the other two conditions taht had similar current densities (192.73 ± 20.35 µA cm^−2^, at 0 mV). These results indicate that S-MFC treating urine can only be scaled-up to an electrode height of around 5–6 cm before the performance is negatively affected.

## Highlights

• S-MFCs with different electrode heights were investigated at the cascade level.
• S-MFCs can be scaled up to 4 cm electrode height without performance decrease.
• S-MFCs cannot be scaled up to 12 cm electrode height without performance decrease.
• The colonisation of the Cathodic biofilms do not exceed 6 cm depth.
• The cathode coverage by the biofilms seems to reflect the S-MFC efficiency.

## 1 Introduction

Microbial fuel cells (MFCs) are of increasing interest because they combine the production of low levels of electricity and the treatment of different types of wastewater: the reduced organic matter contained in wastewater is converted directly into electricity through the metabolic activity of anaerobic electro-active microorganisms [1], [2], [3], [4]. Like in other anaerobic bioreactors, the enriched microbial community degrade the organic matter through a series of anaerobic metabolic reactions. The key characteristic of microbial fuel cells is that the enriched communities performing anaerobic respiration have the capacity to employ an electrode as the end-terminal electron acceptor. During this process, protons, smaller organic molecules and CO_2_ are transferred into the electrolyte. The electrons collected by the anode flow towards the cathode through an external load, whilst protons diffuse towards the cathode half-cell. At the cathode, protons and electrons react through a reduction reaction with an oxidant of a higher redox potential (e.g. oxygen) [5], resulting in the production of current (electron flow). The main advantage of this technology is that it can treat waste streams of various sources (e.g. activated sludge [6], neat urine [7] and others [8], [9]).

In developed urban settings, urine is the source of 75%, 50% and 10% of the nitrogen, phosphorous and COD present in domestic wastewater, respectively, whilst it only amounts to less than 5% of the total volume [10], [11]. Treating this type of waste stream prior to reaching wastewater treatment plants is therefore an attractive solution [11], [12] to increase the energy efficiency of wastewater treatment [7], [13], [14], [15], [16]. To the authors’ best knowledge, the MFC is the only biotechnology able to directly treat neat urine – with no dilution and without succumbing to inhibition due to high ammonium concentrations – and does not require any energy input [17]. A recent study has shown that the chemical oxygen demand (COD) and total nitrogen (TN) loadings can be reduced by 88% (from 5.586 to 0.672 g l^−1^) and 29% (from 4.525 to 3.233 g l^−1^), respectively, at a hydraulic retention time of 44 h [17]. Although these final concentrations would not allow direct discharge into the environment, the removal rates are close to the industrial sector (92% COD and 20% TN reduction) [18].

These reduction rates were obtained with self-stratifying membraneless MFCs (S-MFC) that were recently developed for the treatment of this particular fuel. With microbial fuel cells, usable power levels and enhanced treatment are obtained when a plurality of MFCs are assembled in stacks [19], [20]. For this reason, single MFC units of a stack have to be simple in design and also cost effective. Such an equilibrium between size, design simplicity and cost has been reached with S-MFC [14], [21], [22]. S-MFCs exploit the capacity of microorganisms to structure, in any wet environment, horizontal microenvironments characterised by specific bio-chemical conditions (i.e. redox state of chemical elements, type of dominating metabolic activity) [23]. Here, the S-MFCs exploits this phenomenon in a urine column with the cathodes being placed in the upper oxidised layers and the anodes being placed in the lower reduced layers. The main advantage of this design is that it allows having a plurality of vertical electrodes. Hence, such design authorities the build modules that can be scaled-up in width and length (i.e. more vertical electrodes and longer electrodes, respectively) with no performance losses [14], [17].

**Fig. 1:**
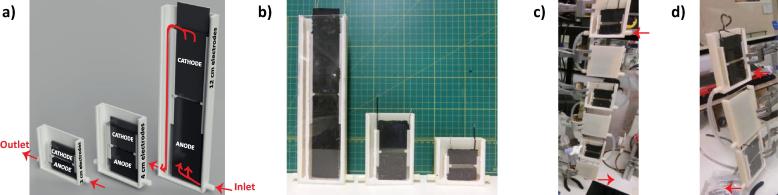
Illustration of the S-MFCs set-ups employed in the present study. 3D CAD models (a) of the three tested conditions (2 cm, 4 cm, 12 cm); the red arrows shows the inlets, the outlets and the flow direction of the fuel. In (b) the picture shows an assembled example of each of the three tested MFC-size. In (c) and (d) the pictures show one of the triplicates assembled cascades: 6 reactors for the 2 cm condition (c) and 3 reactors for the 4 cm condition (d). The 12 cm condition consisted of a single S-MFC. Fresh fuel was pumped in the top MFCs and then cascading to the underneath downstream ones. (For interpretation of the references to colour in this figure legend, the reader is referred to the web version of this article.)

Due to the nature of how this particular fuel is collected, any implementation at ground level would benefit from having shallow units. Until now however, all the studies on S-MFCs have employed units with comparable heights (i.e. ≈10 cm urine column depth; 4.5 cm electrode height) [14], [17], [21], [22], [24]. Although S-MFCs can be scaled in width and length with negligible power density losses, the height scalability of this type of MFC is yet unknown. Hence, the present study reports on the height scalability of S-MFCs mounted with electrodes of 2 cm, 4 cm and 12 cm heights. In these bioreactors, the cathode and anode electrodes had the same height with 3 mm distance from each other. Here, the S-MFC comprised only a single cathode placed above a single anode. The experimental set-up comprised triplicate cascades of either 6 S-MFC, 3 S-MFC and 1 S-MFC, for the 2 cm, 4 cm and 12 cm conditions, respectively (see Fig. 1). Under these conditions, all cascades had the same hydraulic retention time (HRT), total electrode surface area and total displacement volume. The cathodes were based on a carbonaceous-based catalyst pressed over a stainless steel (grade 316) current collectors, and the anodes were made from carbon veil. Once inoculated, the S-MFCs were fed by a continuous flow of urine and maintained under potentiostatic conditions (400 mV). When steady-state was reached, the electrochemical properties were investigated.

## 2 Material and methods

### 2.1 Reactors construction and operation

The S-MFCs employed had the same structure as previously described [14], [22], [25]. However, in the present study each S-MFC comprised a single cathode placed 3 mm above a single anode, both immersed in the same electrolyte. Three reactor sizes have been built with the parameters summarised in Table 1. All the electrodes had the same width (42 mm) and thickness (0.2 cm) with the height being the only variable (2 cm, 4 cm, 12 cm). The embodiment of the S-MFC was 3D-printed (Fortus 250mc, Stratasys Ltd, UK) in Acrylonitrile Butadiene Styrene (ABS-P430 Ivory, Stratasys Ltd, UK) and laterally closed by a 2 mm thick acrylic sheet. Employing 3D-printed structures ensured that the size parameters between each condition were accurately scaled (Fig. 1a and b). The embodiment was designed for the urine level in the reactor to overflow at ¾ of the cathode height that was shown before to be the best operating condition [22]. The reactors were assembled in cascades whereby the outlet of the top S-MFCs feeds into the down-stream S-MFCs. This work focused on the S-MFC sizes, defined by the height of a single electrode between 2 cm and 12 cm, at both the level of an individual S-MFC and the level of a stack of these S-MFCs. To make the comparison meaningful, the total surface area of electrode and total volume between each cascade had to be similar. Hence the only possible sizes to be tested were 2 cm (6-unit cascade), 4 cm (3-unit cascade), 6 cm (2-unit cascade) and 12 cm (single unit). In previous work it was shown that 4.5 cm high electrode condition had similar performances to 2, 3 and 4 cm conditions [22], [25]. Hence, the 12 cm condition was preferred over the 6 cm condition, considered too close from the 4.5 cm conditions. Three cascade conditions were therefore tested in triplicate: the 2 cm condition reactors were assembled as a cascade of 6 units (Fig. 1c); the 4 cm condition reactors were assembled in cascades of 3 units (Fig. 1d); the 12 cm conditions were tested as a single-reactor “cascade”. All cascades had the same total volume, the same total surface area of electrode and the same hydraulic retention time (HRT; Table 1). All the units in a cascade were electrically connected in parallel.

**Table 1:** Parameters of the S-MFCs and the stack configurations.

	Condition	Number	scaling ratio	Electrodes heights	*Electrodes surface area	Volume	Urine column heights	HRT
Single MFC	2 cm	–	1	2 cm	8.4 cm^2^	18 ± 1.0 ml	38 mm	101 min
	4 cm	–	2	4 cm	16.8 cm^2^	36 ± 1.0 ml	73 mm	202 min
	12 cm	–	6	12 cm	50.4 cm^2^	107 ± 1.5 ml	213 mm	606 min
MFC Stack	2 cm	6	–	2 cm	50.4 cm^2^	108 ± 3.0 ml	–	606 min
	4 cm	3	–	4 cm	50.4 cm^2^	108 ± 2.0 ml	–	606 min
	12 cm	1	–	12 cm	50.4 cm^2^	107 ± 1.5 ml	–	606 min

* This is the total projected surface area for both electrodes.

Similar to previous studies [14], [17], [21], [22], [25], the cathodes were made by hot pressing (280 °C) a mixture of activated carbon (AC) and polytetrafluoroethylene (PTFE) (80 wt% AC; 20 wt% PTFE) onto a stainless steel 316 mesh acting as the current collector (8x8 mesh; MeshDirect, UK). Some of the mesh wires were extending out of the cathode to act as the connection point. The AC/PTFE loading on the cathodes was 219 ± 7 mg cm^−2^ (n = 4). The final weight of the single cathodes was of 3.407 ± 0.105 g for the 2 cm conditions (n = 18), 6.856 ± 0.279 g for the 4 cm conditions (n = 9), and 19.330 ± 0.156 g for the 12 cm conditions (n = 3). The anode consisted of carbon veil (20 g m^−2^; Technical Fibre Products Ltd, Cumbria, UK). The total surface area (geometric) of carbon veil was of 60 cm^2^ (150 mm × 40 mm), 120 cm^2^ (300 mm × 40 mm) and 360 cm^2^ (120 mm × 300 mm), for the 2 cm, 4 cm and 12 cm conditions respectively. The carbon veil was then wrapped around the same current collector as the cathodes (stainless-steel 316 mesh), which corresponded to a final projected surface area of 8.4 cm^2^, 16.8 cm^2^ and 50.4 cm^2^, for the 2 cm, 4 cm and 12 cm conditions respectively (Table 1). The final weight of the carbon veil anode was of 0.407 ± 0.031 g for the 2 cm conditions (n = 18), 0.985 ± 0.067 g for the 4 cm conditions (n = 9), and 3.129 ± 0.063 g for the 12 cm conditions (n = 3).

The 9 cascades (triplicate of each conditions) were fed with the same fuel (urine) from a single tank through a multichannel peristaltic pump (Watson & Marlow LTD, UK). The urine was collected daily from a collection tank that pooled anonymously the urine of volunteers. By the time this fuel reached the S-MFCs, a natural partial hydrolysis had occurred and the average pH was 8.5–9.3 and the average solution conductivity was 29 ± 3 mS cm^−1^. The cascades were initially inoculated with urine diluted at 50% (v/v) with effluent from different and mature S-MFCs also fed with urine [25].

### 2.2 Data capture and system characterisation

The cascades were connected, since inoculation and for all the duration of the experiment, to a purpose-built circuitry that maintained each cascade under potentiostatic conditions at 400 mV. This circuit acted as a fixed external Constant Voltage Load, using an operational amplifier (TSZ124IQ4T) with a reference voltage that would load the MFC through an N-channel MOSFET (BSS138) in order to sink current. More detail on the circuitry built is reported in a previous study [25]. This setup allowed to convert the measured current into voltage, which was recorded by an Agilent Data Acquisition System (Agilent LXI 34972A; Farnell, UK), and logged over time every 4 min. The conversion of the measured voltage into the produced current was done by applying the following formula:

(1) $$I=\frac{\left(V_{m}-1.20\right)}{19.8}$$

where *I* is the current in Amperes (A) and *V_m_* is the voltage measured in Volts (V) by the acquisition system. The power *P* in Watt (W) produced by each unit was calculated using the formula, *P = I* × *V*, where *V* is the constant voltage (400 mV) in Volts (V) and *I* is the calculated current using Eq. (1). For comparison purposes, the data were normalised by twice the total surface area of the cathodes since both side of the electrodes were in contact with the electrolyte.

The initial polarisation of the cathodes was performed in urine after being immersed for over 12 h in order to avoid the presence of oxygen adsorbed on the activated carbon surface. The polarisation runs were performed running a linear sweep voltammetry (LSV) using a Biologic potentiostat (SP-50, France) under a three-electrodes configuration with the anodes being used as counter electrodes, the cathodes as the working electrodes and a Ag/AgCl (3 M KCl) as reference electrode being place next to the cathode (geometrical middle of the immersed part) to reduce the ohmic resistance given by the electrolyte (urine). The applied scan rate was slow (0.25 mV s^−1^) in order to avoid overestimation of the performance due to capacitive effect [26], [27]. The LSVs were run between open circuit potential (OCP) and −200 mV vs Ag/AgCl (3 M KCl). Although the systems were composed of multiple unit cascades for the 2 cm and 4 cm conditions, only the cathodes of the first S-MFCs of the cascades were polarised (n = 3 for each conditions). The cathodes and anodes were also polarised using a three-electrodes configuration 4 weeks after inoculation. For the anode polarisation, the cathodes were used as counter electrodes, the anodes as the working electrodes and the Ag/AgCl (3 M KCl) as the reference electrode, which was placed next to the geometrical middle of the anodes. After 4 weeks, once the S-MFCs had matured, overall polarisation curves were performed by LSVs. In this case, a two-electrode configuration (potentiostat Biologic SP-50) was adopted with the reference electrode channel short-circuited with the counter electrode channel. The anodes were the negative electrode and the cathode served as the positive electrode. Also in this case, the scan rate was 0.25 mV s^−1^ [26] and the MFCs were scanned from OCV to 80 mV. Only the first S-MFCs of the cascades were polarised (n = 3 for each conditions), unless otherwise stated. Prior the polarisation experiments, the S-MFCs were left under open circuit conditions (OCV) for 1 h. The data were normalised by twice the projected surface area of the cathodes since both faces of the electrodes were in contact with the electrolyte therefore contribution to the production of useful electricity.

## 3 Results and discussion

### 3.1 Cathode polarisation curves in untreated urine

The cathodes were initially characterised in urine from the collecting tank as electrolyte (i.e. partially hydrolysed, pH = 8.4; EC = 29.46 mS cm^−1^). The polarisation experiments were run in a three-electrode configuration, as described in Section 2.2, on the first S-MFC of each cascade. The hypothesis was that since the wet surface area of the electrodes were proportionally scaled between the three tested conditions, the displayed current density should be the same. Hence, the oxygen reduction reaction (ORR) rate should be proportional to the amount of wet surface area of cathode.

**Fig. 2:**
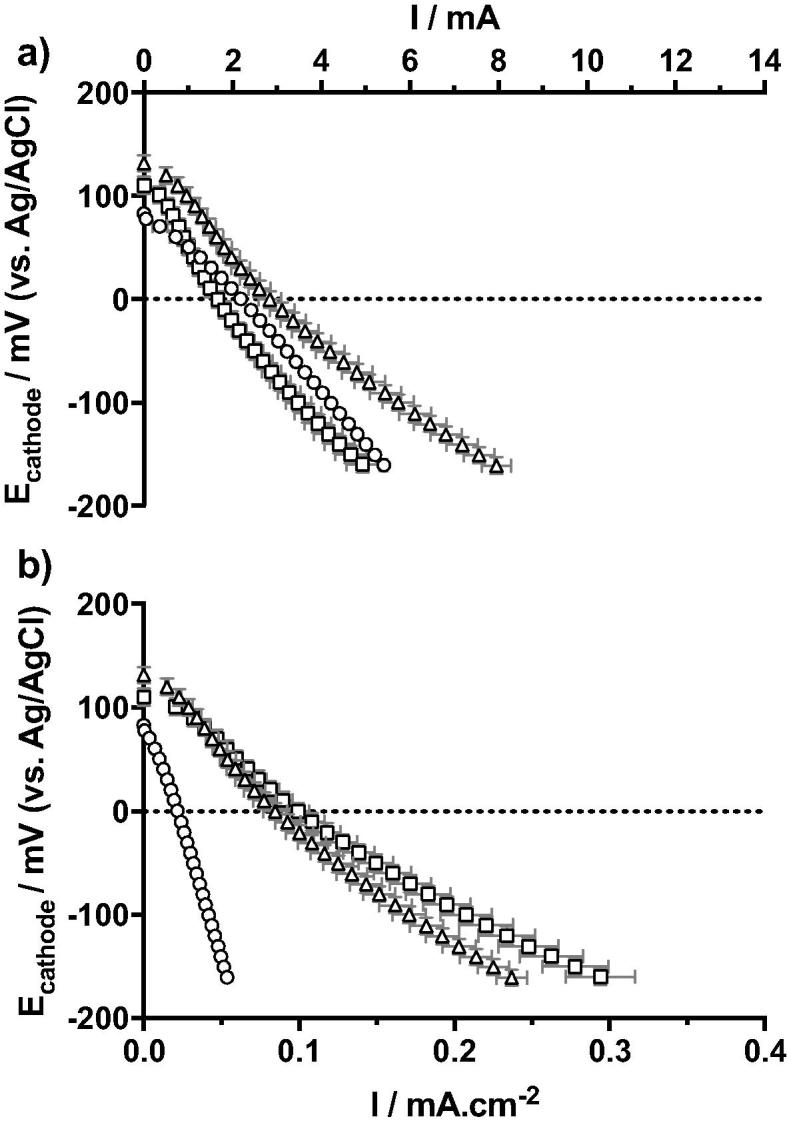
Polarisation curves of the cathodes in untreated and partially hydrolysed urine (pH = 8.06; EC = 14.86 mS cm^−1^) prior to inoculation (□ 2 cm; △ 4 cm; ○ 12 cm). (a) shows the absolute current, whilst (b) shows the current normalised by the total cathode surface area. Error bars stand for the standard deviation of the triplicates.

As the OCP is a thermodynamic characteristic that is independent from the size of electrodes, the open circuit potential (OCP) should be the same for all tested configurations. In fact, Fig. 2 shows similar OCPs measuring 108 ± 24 mV (vs Ag/AgCl). Particularly, the 12 cm condition had the lower value of 83 ± 4 mV (vs Ag/AgCl) while the 2 cm and 4 cm conditions had slightly higher value of 110 ± 9 mV (vs Ag/AgCl) and 131 ± 8 mV (vs Ag/AgCl), respectively). The slightly lower OCPs of the 12 cm cathodes might be due the lower oxygen diffusion from the surface of the water column (air/liquid interface) to the bottom part of the cathode. Although the cathode surface area of the S-MFC mounted with 12 cm electrode is either three times larger (vs 4 cm conditions) or six times larger (vs 2 cm conditions), the current density at 0 mV vs Ag/AgCl (0.0217 ± 0.0004 mA cm^−2^) was four times lower than the 2 cm and 4 cm conditions, 0.0993 ± 0.0072 mA cm^−2^ and 0.0847 ± 0.0078 mA cm^−2^, respectively. These results further support the hypothesis that in the S-MFCs mounted with 12 cm electrodes aerobic and anaerobic zones were forming over the depth of the electrode generating mixed-potentials and therefore limiting the ORR. Conversely, the 2 cm displayed slightly higher but comparable current densities at 0 mV (vs Ag/AgCl) compared to 4 cm conditions, with differences quantified in less than 10%. These results are in line with previous studies on the scalability of individual S-MFC [22], [25].

### 3.2 Temporal behaviour of the S-MFC cascades

After the initial cathode polarisation, cascades were mounted in triplicate with 6 units per cascade for the 2 cm condition, 3 units per cascade for the 4 cm condition and a single unit for the 12 cm condition. All the S-MFCs composing each cascade were electrically connected in parallel. Also, all cascades had the same total volume (108 ± 2 ml), the same total surface area of cathode and anode (50.4 ± 1 cm^2^ and 360 ± 1 cm^2^, respectively) and the same HRT of 606 min (Table 1). Each cascade was then run under potentiostatic condition at 400 mV.

**Fig. 3:**
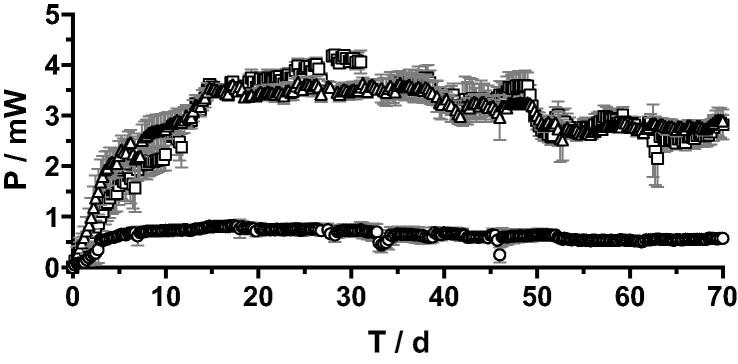
Temporal absolute overall power output of the 3 tested cascades (□ 2 cm; △ 4 cm; ○ 12 cm conditions). All S-MFC in each cascade were electrically connected in parallel. Error bars stand for the standard deviation between triplicates.

**Fig. 4:**
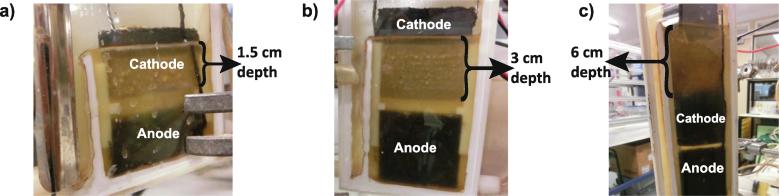
Pictures of the developed cathodic biofilm in the tested S-MFCs 30 days after inoculation. Picture of one of (a) the 2 cm conditions, (b) the 4 cm conditions, and (c) the 12 cm conditions.

Two different groups can be observed through the 60 days run (Fig. 3). On one hand the cascades with 2 cm and 4 cm tall electrodes showed similar power outputs levels (3.61 ± 0.21 mW between day 15 and 40). On the other hand, the 12 cm conditions displayed much lower power output over the entire length of the temporal run (0.66 ± 0.12 mW between day 15 and 60). The power level of these S-MFC mounted with 12 cm tall electrodes only represent 18% of what the two other conditions produced. At the inoculation phase, up to day 3, all tested conditions behaved similarly. However, after day 3, the power production of the 12 cm conditions plateaued whilst the power produced by 2 cm and 4 cm cascade continue increasing similarly, up to day 15 when steady state was reached (Fig. 3). These results indicate that a factor was limiting the performances of the 12 cm S-MFCs. The results from the initial cathode polarisation tend to indicate that the limited power production of the 12 cm conditions could be linked to the limited ORR in the cathodic layers. Moreover, the hypothesis that the ORR is limited in the 12 cm conditions is further supported by the observed development of the cathodic biofilms (Fig. 4).

On the S-MFCs mounted with 12 cm electrodes, the cathodic biofilm does not cover the totality of the cathode. The biofilm only colonises the cathode up to a depth of 5.5–6.0 cm depending on the triplicate observed (Fig. 4c). Conversely, the 2 cm and 4 cm conditions displayed a complete colonisation of the cathode by the cathodic biofilm. The development of this cathodic biofilm could serve as a indicator of the efficiency of the electro-bioreactor’s design. Since the cathodic biofilm in the 12 cm conditions could not extend further than 5.5–6.0 cm in depth, this implies that the redoxcline cannot be established beyond that depth, in S-MFCs treating urine. Interestingly, as mentioned above, diverse environmental conditions such as aerobic and anaerobic might occur along the electrode depth creating mixed potentials and lowering the cathode and the overall performance. These results lead to the observation that S-MFCs cannot be scaled-up in size beyond a cathodic immersion depth of 5.5–6.0 cm. Nonetheless, the cascades of S-MFCs mounted with either 2 cm tall or 4 cm tall electrodes displayed similar power outputs (Fig. 3), hence, confirming that S-MFC can be scaled between 2 cm and 4 cm tall electrodes without performance losses. This hypothesis further support a previous study on the miniaturisation of S-MFCs [25]. However, in the present study the scalability is observed at the cascade scale: A cascade of 6 S-MFCs with 2 cm tall electrodes produce as much overall power as a cascade of 3 S-MFCs with 4 cm tall electrodes.

### 3.3 Characterisation of the individual S-MFCs and the assembled cascades

Once all cascades reached steady state, at day 40 (Fig. 3), the electrodes of each first S-MFC of each cascade were characterised through a linear sweep voltammetry experiment ran in a three-electrodes configuration (see Section 2.2). The polarisation experiments were run after leaving the SSM-MFCs for 1 h under open circuit conditions. In order to minimise the ohmic losses caused by the electrolyte separating the two electrodes [28], the anode and cathode potentials were recorded separately (2 different polarisation experiments) against a Ag/AgCl (3 M KCl) reference electrode placed either next to the middle of the cathode or next to the middle of the anode. During the cathode polarisation, the anode was used as the counter electrode and vice versa during the anode polarisation, the cathode was used as the counter electrode. The following day, the cascades that have all the S-MFCs electrically connected in parallel were characterised through a linear sweep voltammetry experiment ran in a two-electrodes configuration (see Section 2.2).

**Fig. 5:**
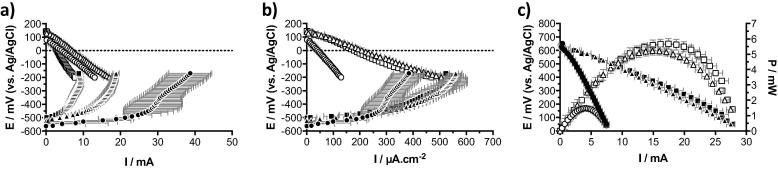
Electrochemical characterisation of S-MFCs. (a) individual electrode characterisation of the first S-MFC of each condition (□ 2 cm; △ 4 cm; ○ 12 cm). (b) current production normalised by the total surface of each cathodes. In (a) and (b), white symbols stand for the cathode curves and the black symbols stands for the anodes curves. (c) polarisation curves of the cascade of each condition. In (c) the white symbols stand for the power curves and the black symbols stands for the potential curves.

As for the other results, the S-MFCs mounted with 12 cm tall electrodes had lower overall performance than the 2 cm and 4 cm conditions (Fig. 5). The open circuit potential (OCP) of the cathode of the 12 cm conditions was half the OCP (67 ± 5 mV) of the 2 cm and 4 cm conditions (128 ± 5 mV; Fig. 5a). Interestingly, the anode’s OCP were increasing with the size of the reactor (2 cm: −498 ± 2 mV; 4 cm: −523 ± 1 mV; 12 cm: −545 ± 6 mV). The anode OCP is mainly influenced by the presence of reduced elements (e.g. organics) and of oxidised elements (e.g. oxygen). The possible explanation for higher potential can be related to the reactor size: the taller is the reactor (12 cm > 4 cm > 2 cm) the further away from the anode is the surface of the electrolyte and the oxygenic atmosphere.

The open circuit voltage (OCV) of all the S-MFCs was similar and measured 623 ± 3 mV, 643 ± 5 mV and 618 ± 10 mV for the 2 cm, 4 cm and 12 cm conditions, respectively. From the perspective of scalability, the results indicate that only the 12 cm conditions differed from the two other conditions. Even though the anode polarisations show that the 12 cm conditions produced more overall current (Fig. 5a), the normalised data for the surface area clearly shown that despite similar behaviour till 300 μA cm^−2^, the current densities of the 12 cm conditions went in diffusion limitation much before compared to the two other conditions, which the limitation was reached around 500 μA cm^−2^ (Fig. 5b). As seen in Fig. 5b, the anodes and cathodes current densities of the 2 cm and 4 cm conditions were comparable, further supporting the scalability of S-MFCs between 2 cm and 4 cm tall electrodes. The limited cathodic biofilm development (Fig. 4c) together with the lower OCP of the cathode (Fig. 5a and b) indicates that part of the 12 cm conditions cathodes is under anoxic condition. The fact of having part of the cathode exposed to both aerobic and anaerobic conditions correspond to a microbial snorkel as defined by Erable et al. [29], thus explaining why at the scale of the 12 cm conditions S-MFCs have limited current output.

The results of the linear sweep voltammetry experiment ran on the cascade indicate that the 2 cm and 4 cm conditions produced at 350 mV similar maximum power outputs of 5.68 ± 0.34 mW and 5.22 ± 0.29 mW, respectively. Conversely, the 12 cm S-MFCs/cascade produced a maximum power of 1.48 ± 0.15 mW at 350 mV (Fig. 5c). These results further support the fact that S-MFCs cannot be mounted with cathodes immersed below a 5.5–6.0 cm depth.

### 3.4 Behaviour of individual S-MFCs within the cascades

**Fig. 6:**
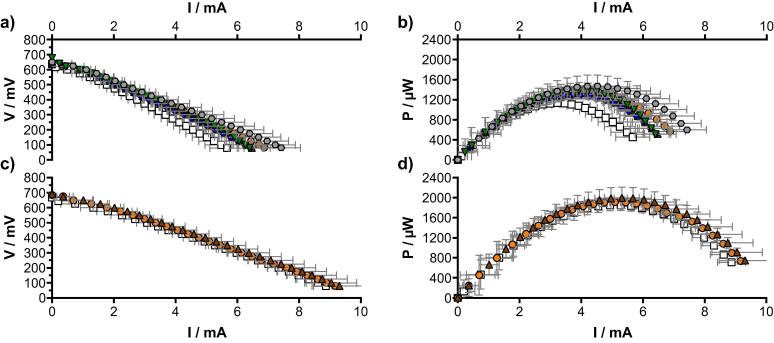
Potential and power curves of each of the S-MFC of the 2 cm conditions (a, b, respectively) and the 4 cm conditions (c, d, respectively), function of their position within the cascade: 1st MFC of the cascade (**□**), 2nd of the cascade (), 3rd of the cascade (), 4th of the cascade (), 5th of the cascade (), 6th of the cascade (). The 2 cm condition stack had 6 MFCs cascading into each other, and the 4 cm cascade had 3 MFCs. Error bars stand for the measured variation between triplicates.

The previous LSVs experiments, in a two-electrodes configuration, have focus on the whole cascade (Fig. 5c). However, beside the 12 cm conditions “cascades” that only comprised a single unit, each of the 2 cm and 4 cm conditions cascades comprised either 6 units or 3 units, respectively. Results have shown that at the cascade level both 2 cm and 4 cm conditions were similar. As shown in a previous study on urine-fuelled MFCs, the size of the reactor and the hydraulic retention time (HRT) affects the performance of both the single unit and the cascade [30]. In the present study and with the described reactors, although at the level of the cascade all parameters were identical, at the level of a single S-MFC the HRT of the 2 cm conditions was 50% shorter than the HRT of a 4 cm conditions (Table 1). Hence, the performance of a single S-MFCs will differ between the two sizes. For this reason, the performance comparison will be made between S-MFCs of the same size, whilst the behaviour of each cell within the cascade will be compared between the two tested sizes. The behaviour of each S-MFC in function of their position within a cascade was thus characterised by a LSV experiment in a two-electrodes configuration. Nonetheless, results show the impact of the position of a S-MFC within a cascade on its electrochemical performance (Fig. 6).

Compared to the downstream S-MFC, the first S-MFCs (Level 1) of the 2 cm condition cascades displayed a lower maximum power transfer point (MPT) of 1134 ± 28 µW (Fig. 6b). The MPT of the downstream S-MFCs ranged from 1300 ± 78 µW (level 4) to 1473 ± 219 µW (level 6). Independently from their positions, all S-MFCs of the 2 cm conditions had the MPT at 350 ± 15 mV (Fig. 6a). Conversely, the S-MFCs with 4 cm tall electrodes displayed similar maximum power transfer point at 375 ± 15 mV (Fig. 6c) (1923 ± 140 µW), independently from their position within the cascade (Fig. 6d). It has to be noted that the average MPT of the first cascade of the 4 cm conditions was slightly lower than the two other cascades (1780 ± 62 µW vs. 1994 ± 62 µW), whereas all three cascades of the 2 cm conditions were more homogenous (1373 ± 32 µW). The impact of the HRT can be seen at two levels. Firstly, compared to the 4 cm conditions’ S-MFCs that had double the HRT, the individual S-MFCs of the 2 cm conditions cascades had higher power densities. Secondly, the level-1 S-MFCs of the 2 cm conditions had a lower MPT than the other downstream S-MFCs. The hypothesis of this lower performance is that either the electrolyte did not yet have the time to be sufficiently reduced and/or the organic content did not have sufficient time to be broken down and be accessible to the electroactive microorganisms. Another hypothesis is the fact that the electrolyte contains oxygen once entering the first S-MFC (2 cm), which is fully consumed and then the electrolyte is completely anaerobic in the 2nd level of the 2 cm S-MFC. The faster flow might affect the self-stratification in the level 1 S-MFC, Moreover, as the flow is slower in the 4 cm condition, it might be possible that the oxygen is consumed within the first S-MFC not affecting the electrochemical output. Another observation is the fact that the S-MFCs from the 2 cm conditions displayed greater variation than the 4 cm conditions, as reflected by the errors bars of the power curves in Fig. 6.

### 3.5 Cathodes polarisation function of the reference depth

**Fig. 7:**
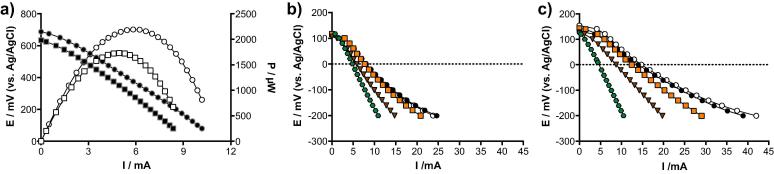
Polarisation of the best (C3, **○**)and less performing (A1, **□**) of the 4 cm conditions S-MFCs in a two-electrode configuration (a). In (a), the black symbols stand for the potential curves and the white symbols stand for the power curves. Cathode polarisation of S-MFC function of the depth of the reference electrode in either the 1st S-MFC of the 1st cascade (A1; b) or in the last S-MFC of the third cascade (C3; c): first with the reference electrode at 2 mm depth (**○**), then at 10 mm depth (), 20 mm depth (), 30 mm depth () and finally again at 2 mm depth (●, control). These are single experimental runs.

During the polarisation experiment of a cathode, the reference electrode slipped from its original position and moved near the surface of the electrolyte. This change in position/depth resulted in an increase of the current production. This observation thus raised the question of the methodology to employ when investigating the electrochemical properties of self-stratifying MFCs. Up to this point, for LSV experiment run in a three-electrodes configuration, the reference electrode was placed next to the geometrical centre of the immersed part of the cathode (Fig. 6). Since the height is a parameter that was investigated in the present study, the depth at which was placed the reference electrode differed from one size to another. Hence, an experiment was set to investigate the impact of the reference electrode depth position on the measured current outputs. LSV experiments in a three-electrodes configuration were performed on the cathodes of two S-MFCs mounted with 4 cm tall electrodes. This experiment was not performed on the 12 cm condition S-MFCs due to the time needed to recover the OCP (≈6 h; data not shown), nor on the 2 cm conditions since only 15 mm of their cathode was exposed to the electrolyte. Due to recovery time between experimental runs, only two S-MFC representatives of the 4 cm conditions were chosen, the S-MFC having the higher power output (the last S-MFC of the third cascade; C3) and the one with the lower power output (the first S-MFC of the first cascade; A1). Polarisation curves and power curves of the S-MFCs A1 and C3 are reported in Fig. 7a. LSV experiment on the cathodes started from the top of the urine column (2 mm) and continued by 10 mm increments, until the bottom of the immersed part of the cathode (30 mm). To confirm that the observed variations were due to the position of the reference electrode and not to the succeeding polarisations, the last run was performed at 2 mm depth and compared to the initial cathode polarisation run at this depth. The results show, for both S-MFCs (A1 and C3), that the current produced by the initial and last 2 mm depth runs are similar (Fig. 7b and c). Hence, the successive polarisations have not impacted the results.

Following results from the LSV experiment run in two-electrodes configuration (Fig. 7a), the cathode of the C1 S-MFC produced more current than the A1 (Fig. 7b and c). Nonetheless, both S-MFCs showed the same response to the increase of the reference electrode depth: the more the reference electrode was immersed in the electrolyte, the less current was produced by the cathodes. This observation implies that the ORR rate was more sluggish in the lower part of the cathode, compared to the ORR rate of the upper part of the cathode. This difference in the ORR rates can be justified by the bio-physico-chemical stratification of the urine column occurring all along the cathode and therefore that the system is more complex than originally thought. This could explain why no oxygen has been measured in the lower part of the cathodic layer [14]. Interestingly, such phenomenon was not observed for the anodes, which is assumed to be completely anaerobic. These results imply that when reporting the performance of the cathode in S-MFCs, the positioning of the reference electrode should be carefully evaluated and therefore also the results interpretation because diverse environmental conditions (aerobic, anaerobic and anoxic) might occur within the same electrode.

### 3.6 Outlook and future work

This work is based on scaling up self-stratifying MFCs and in order to understand the limit in height, 2 cm (6-unit cascade), 4 cm (3-unit cascade) and 12 cm (single unit) were tested. It was noticed that, during over 70 days of operations, the cathodic biofilms colonisation front stops roughly after 5 cm and roughly before 6 cm. It was therefore found that this was the critical condition and further investigation around that specific height should be carried out in further investigations. Experiments conducted on 12 cm height cathode showed mixed potentials occurring and potential readings and conditions different in function of the position of the reference electrode within the electrolyte. Further investigation should also been driven towards: (i) the identification of bacterial profile within the column of urine along the cathode electrode; (ii) measurement of the oxygen profile along the column for identifying the concentration of oxygen and correlate it with the bacterial species.

As microbial fuel cell is a low current/power producing technology, the reduction and limitation of cost is imperative, therefore the choice of the electrode is somehow imposed. In our case, carbon veil was used as anode electrode material. This material is relatively cheap and guarantee high surface area for biofilm attachment and development [31], it provides the possibility of perfusion limiting or neglecting diffusion problems [32], it was shown to be durable for long terms operations [21] and has good electrical conductivity and resistance to corrosion and harsh environments [21]. The properties of this material as well as other carbonaceous-based electrodes can be improved by doping the surface with carbonaceous powder [33], functional groups [34], active polymers [35] or metals nanoparticles [36]. All these functionalisations produces changes on the surface such as: (i) enhance the electrical conductivity of the carbon substrate; (ii) modify surface roughness inserting anchoring point on the surface; (iii) increment the hydrophillicity of the surface helping the biofilm attachment and growth, (iv) act as mediator improving the electron transfer to the electrode surface [37].

In parallel, also the cathode used in this work is a traditional AC/PTFE mixture pressed over a stainless steel mesh. Commercially available AC possesses high surface area and they are available in large quantity at low cost. AC is a good catalyst mainly because of its high porosity being the neutral media limited significantly by the pH and the very low concentration of H^+^ and OH^−^ that are reagents during ORR [38]. Due to its high surface area, AC does not exhibit high electrical conductivity that can be enhanced by the addition of carbon black, graphene or other carbonaceous materials [39]. Another way of enhancing the ORR catalysis in neutral media is utilising platinum (Pt) or Pt-based catalysts but the excessive cost and the low durability decrease their extensive utilisation for this type of fuel cells. At last, platinum-group-metal-free (PGM-free) catalysts based on nitrogen coordinated with transition metals such as Mn, Fe, Co and Ni have captured the attention of scientists worldwide [39]. The addition of a small quantity of these catalysts can boost the performance importantly without affecting heavily the overall cost [40]. Focusing on the cathode utilised for this experimentation, cost might be reduced without influencing negatively the output by decreasing the AC/PTFE loading on the current collector.

Generally, the advancement in electrode materials properties certainly would lead to an increase in performance output but it would certainly also increase the overall cost of the system. Therefore critical attention has to be devoted on this topic probably exploring a detailed cost-benefit analysis.

## 4 Conclusion

The scalability of individual bioreactors is important aspect of the research in the microbial fuel cells field. It is necessary to define the size of the individual bioreactors to maintain maximum efficiency when assembling them into stacks that are aimed at implementing the technology under real conditions of use. In this perspective, the results presented here further define the scalability range of S-MFC treating urine. A previous study has shown that the lower scalability limit on the individual bioreactor was between 1 cm and 2 cm electrode height. Here, results suggest that single S-MFC bioreactors could be scaled up to 5–6 cm electrode height before having decreased electrochemical performances. This hypothesis is supported by the similar electrochemical performance of either the single bioreactors or the cascades of S-MFC under 2 cm and 4 cm conditions. Conversely, the S-MFCs under 12 cm conditions have shown decrease performance. This poor performance was shown to be linked to the cathodic biofilm development. In S-MFC treating neat urine, the cathodic biofilm only colonised the upper 5–6 cm of the cathode. This indicates that the cathode has a mixed redox potential between the top and bottom part of the cathode, with the upper part being more oxidised than the lower part which was more reduced. These results imply that if the size of the individual reactor should be increased, the design should be modified to match these redox differences and minimise their impact on performance.

## Declaration of Competing Interest

The authors declare that they have no known competing financial interests or personal relationships that could have appeared to influence the work reported in this paper.
